# Systematic differences in effect estimates between observational studies and randomized control trials in meta-analyses in nephrology

**DOI:** 10.1038/s41598-021-85519-5

**Published:** 2021-03-17

**Authors:** Miho Kimachi, Akira Onishi, Aran Tajika, Kimihiko Kimachi, Toshi A. Furukawa

**Affiliations:** 1 Department of Healthcare Epidemiology, School of Public Health in the Graduate School of Medicine, Kyoto University, Yoshida-Konoe-cho, Sakyo-ku, Kyoto, 606-8501 Japan; 2 Department of Rheumatology and Clinical Immunology, Kobe University Graduate School of Medicine, Hyōgo, Japan; 3 Department of Psychiatry, Kyoto University Hospital, Kyoto, Japan; 4 Department of Health Promotion and Human Behavior, School of Public Health in the Graduate School of Medicine, Kyoto University, Kyoto, Japan

**Keywords:** Nephrology, Kidney, Kidney diseases, Epidemiology

## Abstract

The limited availability of randomized controlled trials (RCTs) in nephrology undermines causal inferences in meta-analyses. Systematic reviews of observational studies have grown more common under such circumstances. We conducted systematic reviews of all comparative observational studies in nephrology from 2006 to 2016 to assess the trends in the past decade. We then focused on the meta-analyses combining observational studies and RCTs to evaluate the systematic differences in effect estimates between study designs using two statistical methods: by estimating the ratio of odds ratios (ROR) of the pooled OR obtained from observational studies versus those from RCTs and by examining the discrepancies in their statistical significance. The number of systematic reviews of observational studies in nephrology had grown by 11.7-fold in the past decade. Among 56 records combining observational studies and RCTs, ROR suggested that the estimates between study designs agreed well (ROR 1.05, 95% confidence interval 0.90–1.23). However, almost half of the reviews led to discrepant interpretations in terms of statistical significance. In conclusion, the findings based on ROR might encourage researchers to justify the inclusion of observational studies in meta-analyses. However, caution is needed, as the interpretations based on statistical significance were less concordant than those based on ROR.

## Introduction

Randomized controlled trials (RCTs) provide high-level evidence because they can minimize threats to internal validity. However, it is difficult to conduct RCTs in certain situations, such as with participants with serious complications, interventions with ethical constraints (e.g. surgical procedures) and serious adverse effects^1–4^. In particular, RCTs in nephrology have been limited because patients with kidney diseases generally have a number of complications^5–9^. When the number of available RCTs is insufficient, meta-analyses restricted to RCTs can be misleading^10,11^. Authors of such meta-analyses might then be justified in including observational studies^3,12^. Observational studies can reflect real world practices and have superior generalizability in comparison with RCTs under ideal conditions. However, the GRADE system states that nonrandomized studies constitute only a low level of evidence due to many threats to internal validity^13^. Discrepancies in the findings between observational studies and RCTs can be caused by differences in sample size, confounding factors, and biases such as selection bias, publication bias, and follow-up period^14,15^. In particular, unmeasured confounding factors can hamper causal inferences between the exposure and outcome^16,17^. Despite such controversy, several studies reported that risk estimates obtained from meta-analyses of observational studies did not differ from those from RCTs^14,18,19^. However, the evidence has not been sufficiently established in nephrology. Further, recent meta-analyses which compared observational studies with RCTs based their conclusions on the ratio of odds ratios (ROR) between the pooled OR derived from observational studies and those derived from RCTs, whereas most clinical studies generally interpret efficacy based on statistical significance^18–22^.

Therefore, in the present study, we aimed to (1) assess the trends and characteristics of systematic reviews of observational studies in nephrology in the past decade; and (2) quantify systematic differences in effect estimates between observational studies and RCTs in meta-analyses using two statistical methods: ROR, and discrepancies in statistical significance between the two study designs among meta-analyses combining observational studies and RCTs.

## Methods

### Literature search and selection of studies

The literature searches were conducted in January 2017 using EMBASE and MEDLINE. We searched studies published from January 2006 to December 2016 with no language limitation. The search strategy was developed with the assistance of a medical information specialist and included key words related to 'observational study', 'systematic review', and 'kidney disease' (see Supplement Table 1). Search terms relevant to this review were collected through expert opinion, literature review, controlled vocabulary—including Medical Subject Headings (MeSH) and Excerpta Medica Tree—and a review of the primary search results. The titles and abstracts were screened independently by two authors (M.K, K.K) and were excluded during screening if they were irrelevant to our research question or duplicated. Studies suspected of including relevant information were retained for full text assessment using inclusion and exclusion criteria. If more than one publication of one study existed, we grouped them together and adopted the publication with the most complete data. The present study was conducted according to a protocol prospectively registered at PROSPERO (CRD42016052244).

### Evaluation of the characteristics of the systematic reviews of observational studies

We included systematic reviews of all comparative observational studies in nephrology to assess the trends and characteristics of systematic reviews of observational studies in nephrology in the past decade. We included systematic reviews published from 2006 to 2016 to assess the influence of reporting assessment tools including PRISMA (Preferred Reporting Items for Systematic Reviews and Meta-analyses)^23^ published in 2009 and the risk of bias (RoB) tools including the Newcastle–Ottawa Scale (NOS)^24^ in 2007 and the Cochrane Risk of Bias Assessment Tool for Non-Randomized Studies of Interventions (ACROBAT-NRSI)^25^ in 2014.

We selected studies of kidney disease based on the following two criteria:

1. We included studies on participants with kidney diseases. Kidney diseases were defined as diseases that occurred in the renal parenchyma, such as acute or chronic kidney injury, kidney neoplasms, and nephrolithiasis, based on the MeSH search builder of the term 'Kidney Diseases'. Studies were excluded if they had participants with extra-renal diseases including ureteral, urethral, and urinary bladder diseases.
2. We included studies with primary outcomes related to kidney diseases. We used the same definition of kidney diseases as above. We excluded studies in which kidney diseases were treated as a composite outcome (e.g. composite outcome of kidney, pancreas, and liver cancers).

We described the characteristics of systematic reviews of observational studies as follows:

1. The number of published systematic reviews of observational studies per year
2. Country of first author’s institution
3. Designs of observational studies
  Cohort studies included prospective and retrospective cohort studies
  Case–control studies included ordinary and nested case–control studies.
4. Number of primary studies included in each review
5. Cause of kidney injury
6. Funding source
  Included support from both public institutions and industrial firms
7. Whether each review assessed the RoB
8. Whether each review performed a reporting assessment
  Reporting assessment tools were PRISMA^23^, MOOSE (Meta-analyses of Observational Studies in Epidemiology)^26^, and QUAROM (The Quality of Reporting of Meta-analyses)^27^. STROBE (Strengthening the Reporting of Observational Studies in Epidemiology)^28^, CONSORT (Consolidated Standards of Reporting)^29^, and others were excluded.

### Comparison of effect estimates between observational studies and RCTs in meta-analyses combining both types of study

To compare the effect estimates between study designs, we focused on meta-analyses which combined observational studies and RCTs and compared two specific interventions. We included non-randomized studies, such as cohort, case–control, cross-sectional, and controlled trials that use inappropriate strategies of allocating interventions (sometimes called quasi-randomized studies), as observational studies^30^. We included all studies related to the above-mentioned kidney diseases and did not focus on specific comparative studies. We compared the effect estimates obtained from observational studies as a measurement of exposure with those from randomized studies as a measurement of control in meta-analyses combining both types of studies. We expressed the quantitative differences in effect estimates for primary efficacy outcomes between study designs, taking the ROR^31^. Further, we assessed discrepancies in statistical significance between study designs. The absence of discrepancies, which represents agreement between efficacy and effectiveness, was defined as follows: (1) both study types were significant with the same direction of point estimates, and (2) both study types were not significant. In contrast, the presence of discrepancies was defined as follows: (1) one study type was significant while the other type was not significant, and (2) both study types were significant, although the point estimates had the opposite direction^24^. The assessment of the methodological quality of these meta-analyses combining both types of studies was performed using the AMSTAR (assessment of multiple systematic reviews) 2 appraisal tool^32^.

### Data extraction

Two authors (M.K., K.K.) independently performed full screening to capture the trends and characteristics of systematic reviews of observational studies in the past decade. Three authors (M.K., A.O., A.T.) independently extracted the relevant data, such as the number of events or non-events, to compare the effect estimates between observational studies and RCTs in meta-analyses combining both types of studies. In addition, two authors (M.K., A.O.) independently graded each review for overall confidence as high, moderate, low, and critically low using the AMSTAR 2 tool.

### Statistical analyses

We described the baseline characteristics of systematic reviews of observational studies using means (standard deviation [SD]) for continuous data with a normal distribution, medians (interquartile range [IQR]) for continuous variables with skewed data, and proportions for categorical data.

For the comparison of effect estimates between observational studies and RCTs in meta-analyses combining both types of studies, we estimated the ROR of the pooled OR obtained from observational studies versus those from RCTs. If an OR was not reported in a review, we recalculated the OR by extracting the number of events and non-events in both the intervention and control groups from a review or the primary study itself. If the number of events or non-events was 0, we added 0.5 to all cells of each result^30^. If we could not find the number of events or non-events from a review or primary articles to calculate the OR, we substituted original outcome measures, such as relative risks or risk ratios (RR), and hazard ratios (HR), instead of OR^21,31^. In addition, standardized meanfrom a review or the primary study itself. If the number differences (SMD) and mean differences (MD) were converted to ORs based on a previous study^33^. The standard errors (SEs) and 95% CI were calculated in accordance with previous studies^22,31^. Further, if the reviews did not report effect sizes separately for two designs, we synthesized the results obtained from primary articles. If positive outcomes such as survival were adopted, the OR comparing the intervention with control were coined. In addition, if ordinary or older interventions were included in the numerator of the OR, those OR were also coined. If several outcomes were reported, we used the first outcome that was described in the paper.

We estimated the differences in the primary efficacy outcomes between study designs by calculating the pooled ROR with the 95% CI using a two-step approach^34^. First, the ROR was estimated with the OR obtained from observational studies and RCTs in each review using random effects meta-regression. Second, we estimated the pooled ROR with the 95% CI across reviews with a random-effects model. Further, we performed sensitivity analysis using fixed effect model. If the ROR was more than 1.0, this would indicate that the OR from observational studies were larger than those from RCTs^22,31^. Heterogeneity was estimated using I^2^ test^30^. I^2^ values of 25%, 50% and 75% represent low, medium and high levels of heterogeneity.

Further, we examined the association between discrepancies in statistical significance of each design in accordance with above-mentioned definitions and risk factors using a multiple logistic regression model, adjusted for differences in the number of primary articles between study designs, publication year, countries of first authors, pharmacological intervention, adjustment for confounding factors, and methodological quality of systematic reviews based on rating overall confidence of AMSTAR 2 tool.

All statistical analyses were performed using STATA 16.0 (StataCorp LLC, College Station, TX, USA).

## Results

### Study flow diagram

**Figure 1:**
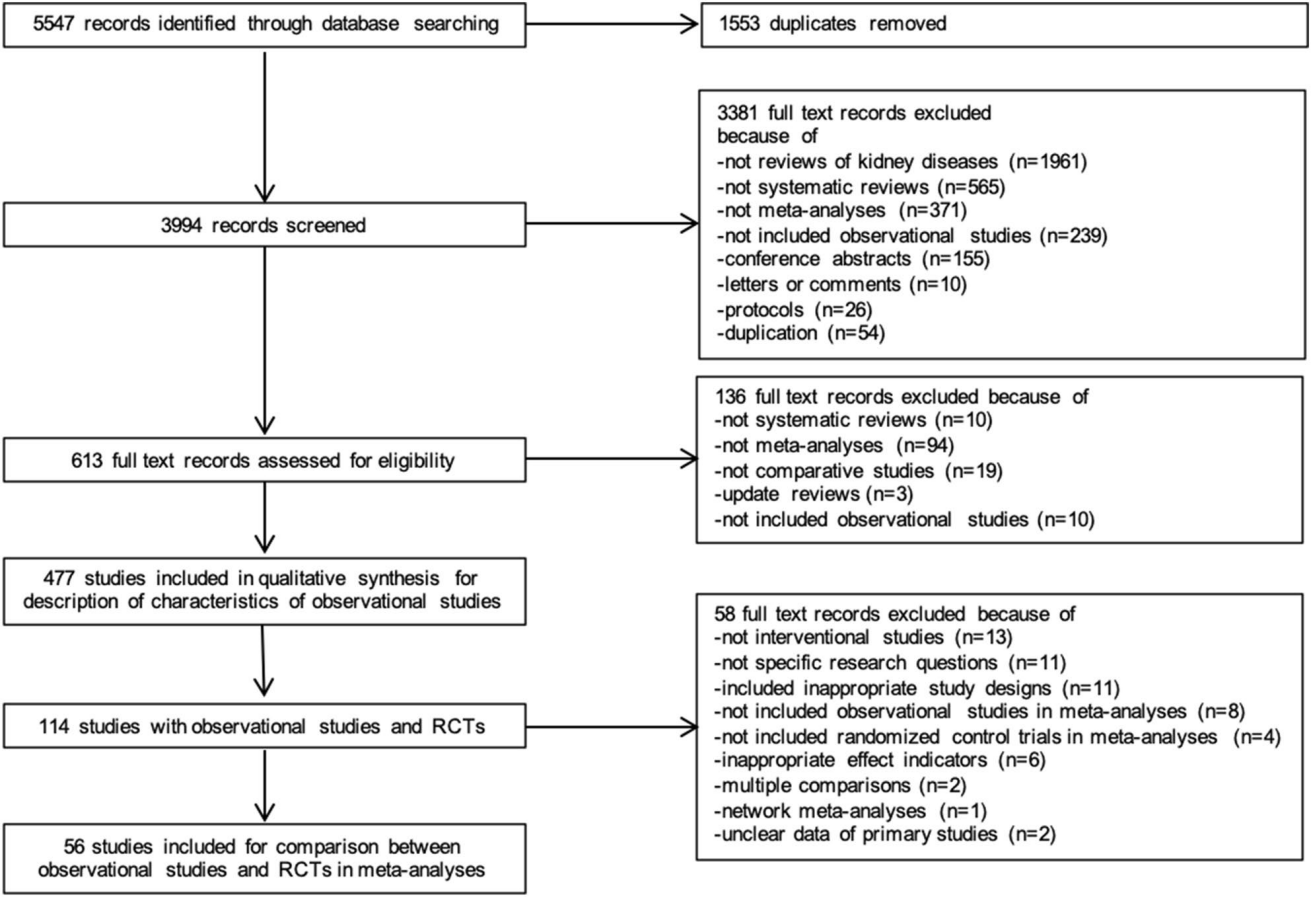
Study flow diagram and study selection process.

The PRISMA flow diagram (see Fig. 1) shows the study selection process. Of 5,547 records identified through database searching, we screened the titles and abstracts of the 3994 records remaining after removing duplicates and ultimately obtained 613 records. After a full-text review, we included a total of 477 records for the description of characteristics of systematic reviews of observational studies. Further, of the 114 records that combined both observational studies and RCTs, 56 were eligible for the evaluation of quantitative systematic differences in effect estimates of meta-analyses between observational studies and RCTs (see Supplement Table 2).

### Trends over the past decade and description of study characteristics

We summarized the baseline characteristics of 477 nephrology systematic reviews of all comparative observational studies (see Table 1). The number of systematic reviews of observational studies in nephrology increased 11.7-fold between 2006 and 2016. In particular, the number of publications from China, as well as the United States of America (USA) and European countries increased (see Supplement Table 3). As shown in Table 1, most of the reviews dealt with topics related to therapies for patients with acute kidney injury, malignancy, end-stage renal diseases, and renal transplantation, aside from basic research. As for the eligible designs of observational studies, 67.1% of records included cohort studies and 33.8% included case–control studies. Of the 82 reviews related to basic research, 75 (91.5%) included case–control studies. Case series and before-after studies without comparisons were excluded in many studies. NOS was the most frequently used tool for assessing the risk of bias. ACROBAT-NRSI was used in only 0.8% of records.

**Table 1:** Baseline characteristics of the 477 included systematic reviews of observational studies.

Characteristics	
Number of primary studies	13 (9 to 21)
**Cause of kidney injury**	
Renal tumor	75 (15.7%)
End stage renal disease	68 (14.3%)
Renal transplant	39 (8.2%)
AKI·CIN	50 (10.5%)
Nephrotoxin	9 (1.9%)
Chronic glomerulonephritis	11 (2.3%)
Diabetic nephropathy	14 (2.9%)
Kidney stone	14 (2.9%)
Basic Research (genetic, molecular, others)	82 (17.2%)
Children or pregnant women with renal diseases	9 (1.9%)
CKD (complication, risk factor, therapy, outcome research, others)	99 (20.8%)
Others (RDN, renal artery, others)	7 (1.5%)
**Included studies**	
RCT	114 (23.9%)
Clinical trials (nonrandomized studies)	18 (3.8%)
Cohort studies	320 (67.1%)
Case–control studies	161 (33.8%)
Cross sectional studies	52 (10.9%)
Case series	5 (1.1%)
Before-after studies	7 (1.5%)
Unclear observational studies	54 (11.3%)
**Funding support**	
Yes	210 (44.0%)
No	102 (21.4%)
Unclear	165 (34.6%)
**Risk of bias in observational studies**	
NOS	183 (38.4%)
Tool by Hayden et al.^44^	14 (2.9%)
Tool by Downs and Black^45^	13 (2.7%)
Tool by USPSTF/task force^46^	11 (2.3%)
ACROBAT NRSI^1^	4 (0.8%)
Others	38 (8.0%)
Unclear	26 (5.5%)
None	188 (39.4%)
**Reporting assessment**	
Yes	227 (47.6%)
No	250 (52.4%)

Results of continuous variables are shown as the mean (standard deviation) or median (interquartile range).

*AKI* acute kidney injury, *CIN* contrast induced nephropathy, *CKD* chronic kidney disease, *RCT* randomized controlled trial, *NOS* Newcastle–Ottawa Scale, *USPSTF/Task Force* The U.S. Preventive Services Task Force, *ACROBAT-NRSI* A Cochrane Risk of Bias Assessment Tool for Non-Randomized Studies of Interventions.

### Comparison of qualitative systematic differences in effect estimates between observational studies and RCTs in meta-analyses combining both types of studies

Fifty-six meta-analyses combining both observational studies and RCTs were eligible for the analyses. A total of 418 observational studies and 204 RCTs were included, and the median number (interquartile range) per meta-analysis was 7 (2.5 to 10) observational studies and 3 (2 to 5) RCTs. Almost all reviews indicated a critically low quality (see Supplement Table 4).

**Figure 2:**
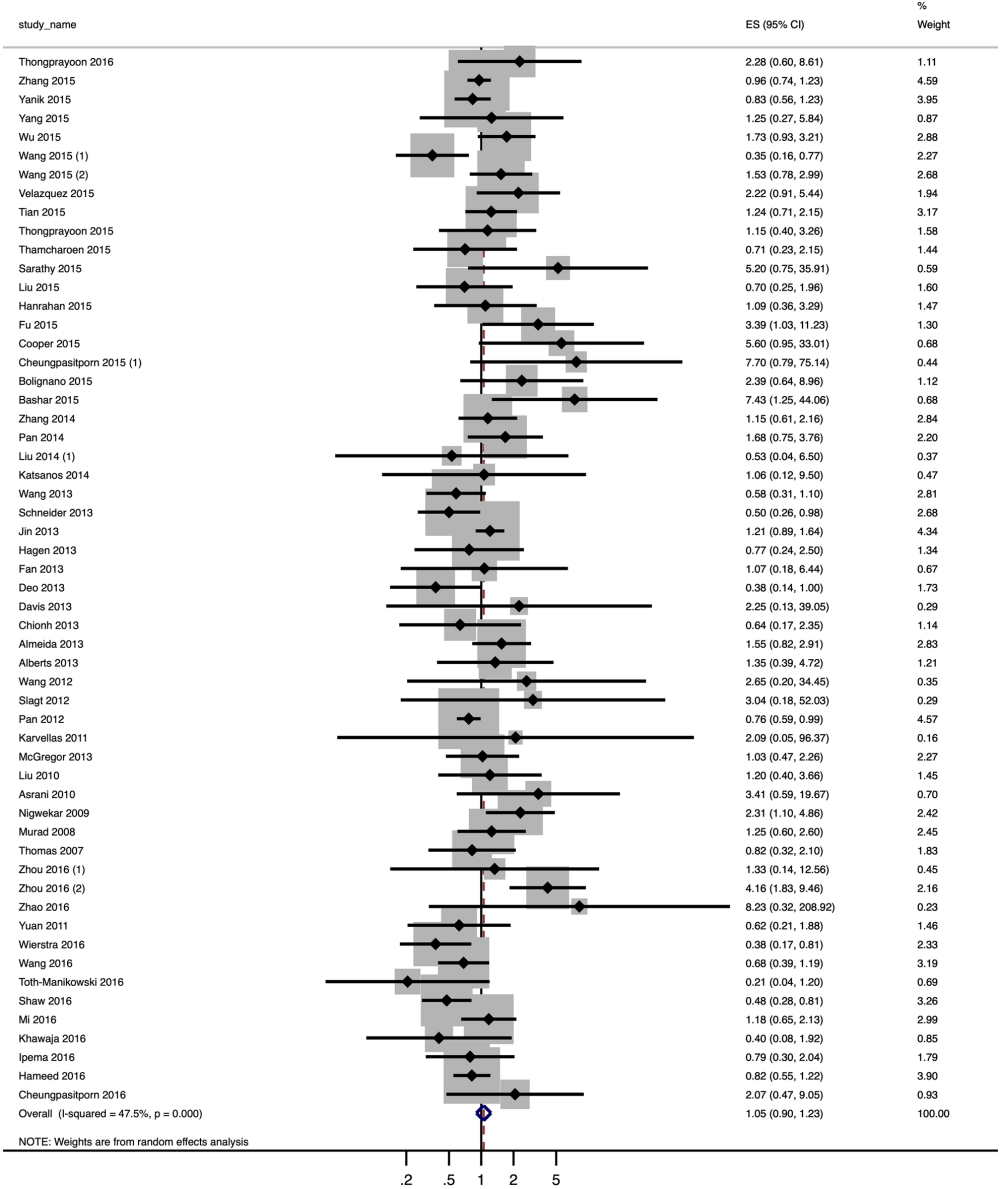
Forest plot of the pooled ROR with the 95% CI to assess systematic differences in the effect estimates between observational studies and RCTs in meta-analyses combining both types of study in nephrology. *ROR* ratio of odds ratio, *CI* confidence interval, *RCT* randomized controlled trial.

We compared the effect estimates of primary outcomes between study designs using ROR with 95% CI. No significant differences were noted in the effect estimates by study designs (ROR 1.05, 95% CI 0.90 to 1.23) (see Fig. 2). There was moderate heterogeneity (I^2^ = 47.5%). Additionally, the result obtained using the fixed effect model was closely similar to that obtained using the random effect model (ROR 0.98, 95% CI 0.89 to 1.07). Of the 56 studies, 2 reviews showed that observational studies had significantly larger effects than RCTs (ROR > 1.0), while 6 showed that observational studies had significantly smaller effects than RCTs (ROR < 1.0). The remaining 48 reviews indicated no significant differences between the study designs.

Of the 56 studies, 29 reviews showed no discrepancy in terms of statistical significance (14 reviews, significant in the same direction as the point estimates; 15 reviews, neither significant), while 27 reviews showed some discrepancy (all 27 studies, one significant and the other not significant). No review showed statistical significance in the opposite direction of the point estimates. Table 2 compares baseline characteristics between the presence and absence of discrepancies. In addition, we explored the factors associated with discrepancies (see Table 3) but found no significant association for any covariate; in particular, we found no differences in the number of papers between observational studies and RCTs (OR 1.10, 95% CI 0.99 to 1.23).

**Table 2:** Baseline characteristics of the 56 included meta-analyses combining both observational studies and randomized control trials.

	Total	No discrepancy (significant in the same direction) (n = 14)	No discrepancy (both non-significant) (n = 15)	Discrepancy (one significant and the other non-significant) (n = 27)
**Number of primary studies**				
Overall studies	11 (6.5 to 14.5)	12 (5 to 15)	7 (4 to 11)	12 (9 to 16)
Observational studies	7 (2.5 to 10)	4.5 (1 to 11)	4 (2 to 8)	9 (5 to 13)
RCTs	3 (2 to 5)	2.5 (1 to 9)	2 (2 to 4)	3 (2 to 5)
**Countries (%)**				
China/Taiwan	21 (37.5)	4 (28.6)	6 (40.0)	11 (40.7)
USA	17 (30.4)	6 (42.9)	3 (20.0)	8 (29.6)
European countries	10 (17.9)	1 (7.1)	5 (33.3)	4 (14.8)
Others	8 (14.3)	3 (21.4)	1 (6.7)	4 (14.8)
**Publication years, n (%)**				
2012 or before	10 (17.9)	1 (7.1)	2 (13.3)	7 (25.9)
2014–2013	15 (26.8)	3 (21.4)	4 (26.7)	8 (29.6)
2015–2016	31 (55.4)	10 (71.4)	9 (60.0)	12 (44.4)
**Cause of kidney injury, n (%)**				
Renal tumor	4 (7.1)	1 (7.1)	2 (13.3)	1 (3.7)
End stage renal disease	11 (19.6)	2 (14.3)	2 (13.3)	7 (25.9)
Renal transplant	7 (12.5)	4 (28.6)	0 (0)	3 (11.1)
AKI·CIN	17 (30.4)	2 (14.3)	4 (26.7)	11 (40.7)
Nephrotoxin	2 (3.6)	0 (0)	2 (13.3)	0 (0)
Chronic glomerulonephritis	1 (1.8)	0 (0)	0 (0)	1 (3.7)
Diabetic nephropathy	1 (1.8)	1 (7.1)	0 (0)	0 (0)
Kidney stone	6 (10.7)	2 (14.3)	4 (26.7)	0 (0)
Children or pregnant women with renal diseases	1 (1.8)	1 (7.1)	0 (0)	0 (0)
CKD (complication, risk factor, therapy, outcome research, others)	6 (10.7)	1 (7.1)	1 (6.7)	4 (14.8)
**Included studies, n (%)**				
Cohort studies	45 (80.4)	11 (78.6)	13 (86.7)	21 (77.8)
Case–control studies	7 (12.5)	1 (7.1)	3 (20.0)	3 (11.1)
Cross-sectional studies	3 (5.4)	0 (0)	1 (6.7)	2 (7.4)
Unclear observational studies	11 (19.6)	4 (28.6)	0 (0)	7 (25.9)
Other non-randomized studies	5 (8.9)	0(0)	2 (13.3)	3 (11.1)
Subjective outcomes, n (%)	56 (100)	14 (100)	15 (100)	27 (100)
Pharmacological interventions, n (%)	18 (32.1)	5 (35.7)	6 (40.0)	7 (25.9)
**Funding support for SR, n (%)**				
Yes	22 (39.3)	10 (71.4)	3 (20.0)	9 (33.3)
No	16 (28.6)	2 (14.3)	7 (46.7)	7 (25.9)
unclear	18 (32.1)	2 (14.3)	5 (33.3)	11 (40.7)
**Risk of bias in observational studies, n (%)**				
NOS	25 (44.6)	9 (64.3)	7 (46.7)	9 (33.3)
Downs and Black	4 (7.1)	0 (0)	1 (6.7)	3 (11.1)
ACROBAT NRSI	1 (1.8)	0 (0)	0 (0)	1 (3.7)
Others	10 (17.9)	1 (7.1)	2 (13.3)	7 (25.9)
Unclear	2 (3.6)	0 (0)	0 (0)	2 (7.4)
None	14 (25.0)	4 (28.6)	5 (33.3)	5 (18.5)
**Existence of reporting assessment, n (%)**	32 (57.1)	7 (50.0)	6 (40.0)	19 (70.4)

Results of continuous variables are shown as the mean (standard deviation) or median (interquartile range).

*RCT* randomized controlled trial, *USA* United States of America, *AKI* acute kidney injury, *CIN* contrast induced nephropathy, *CKD* chronic kidney disease, *NOS* Newcastle–Ottawa Scale, *USPSTF/Task Force* The U.S. Preventive Services Task Force, *ACROBAT-NRSI* A Cochrane Risk of Bias Assessment Tool for Non-Randomized Studies of Interventions.

**Table 3:** Predictors of discrepancies in results between observational studies and randomized control trials.

	OR (95% CI)	p-value
**Differences in number of papers between observational studies and RCTs**	1.09 (0.99 to 1.21)	0.085
**Countries**		
China/Taiwan	1.04 (0.25 to 4.28)	0.96
USA	Reference	
European countries	0.63 (0.10 to 3.89)	0.62
Other countries	1.11 (0.17 to 7.33)	0.91
**Publication year**		
2012 or before	Reference	
2013–2014	0.59 (0.095 to 3.64)	0.57
2015–2016	0.45 (0.087 to 2.34)	0.34
**Pharmacological interventions (vs. none)**	1.12 (0.27 to 4.64)	0.87
**Adjustment for confounding factors**	0.59(0.14 to 2.49)	0.48

Adjusted for differences in the number of primary articles between observational studies and RCTs, publication year, country of first author, and pharmacological intervention.

*OR* odds ratio, *CI* confidence interval, *USA* United States of America.

Further, on comparison of the results of ROR and the distribution of discrepancies of statistical significance, of 48 records (85.7%) that indicated non-significance of the ROR, 20 (35.7%) showed discrepancies in statistical significance (see Table 4).

**Table 4:** Comparison of ROR and discrepancies defined by statistical significance.

	Discrepancy in the interpretations based on statistical significance		
	No discrepancy (significant in the same direction) (n = 14)	No discrepancy (both non-significant) (n = 15)	Discrepancy (one significant and the other non-significant) (n = 27)
**ROR**			
p < 0.05 (n = 8)	1 (1.8)	0 (0)	7 (12.5)
p ≥ 0.05 (n = 48)	13 (23.2)	15 (26.8)	20 (35.7)

*ROR* ratio of odds ratio, number (%).

## Discussion

Our findings indicate that the number of systematic reviews of observational studies in nephrology have dramatically increased in the past decade, especially from China and the USA. Around 60% of reviews assessed the risk of bias, mostly using the NOS. A comparison of effect estimates between observational studies and RCTs in meta-analyses combining both types of studies revealed that the effect estimates from observational studies were largely consistent with those from RCTs. However, when interpreted in terms of statistical significance, almost half of the reviews led to discrepant interpretations.

Observational studies generally have larger sample sizes and better represent real-world populations than RCTs. Nevertheless, confounding factors, especially confounding by indication, often disturb the precise assessment of causal inference and establishment of high levels of evidence^35–38^. The quality of evidence based on observational studies might depend on how confounding factors are controlled. Adjustment using appropriate techniques, including propensity score matching and instrumental variables, are likely to be useful, although these methods cannot completely deal with unmeasured variables^39,40^. However, most of the reviews included in the present study did not mention the implementation of adjustment in detail.

Recently, several risk of bias appraisal tools for evaluating the quality of systematic reviews of observational studies in multiple domains have been developed, including ACROBAT-NRSI^25,41,42^. However, the present study showed that these tools are not yet widely implemented. Most of the studies reported the risk of bias using the NOS, although this has been reported to show uncertain reliability and validity in previous studies^24,43^.

In the present study, we compared the effect estimates between observational studies and RCTs in meta-analyses combining both types of studies using two analytical methods: ROR and discrepancies in statistical significance between the study designs. ROR with a 95% CI revealed that effect estimates were, on average, consistent between the two study designs. These findings would encourage researchers to justify the inclusion of observational studies in meta-analyses. Combining different types of designs in meta-analyses based on the ROR may be reasonable, as improvement in statistical power leads to a more definite assessment if a sufficient number of RCTs cannot be obtained. Further, the degree of guideline recommendations in nephrology is almost always low because evidence from high-quality RCTs is lacking. The increase in evidence derived from the finding that the effect estimates of observational studies are similar to those of RCTs might lead to an improvement in the quality of guidelines in nephrology.

However, with regard to the interpretation of the findings, almost half of records showed discrepancies in statistical significance between the study designs. Further, 35.7% of records indicated disagreement in judgement between the two analytical methods. Therefore, the findings should be interpreted with care, as inconsistent findings due to the modification of analytical methods might reflect poor internal validity between the study designs. In addition, the present study failed to identify systematic review-level factors associated with discrepancies in statistical significance, including differences in the number of primary articles between study designs and the implementation of adjustment with confounding factors. Future studies should explore risk factors at the primary study level.

Several limitations of our study should be mentioned. First, it is possible that we failed to include several gray-area studies or smaller studies, Albiet that we performed a comprehensive search. Second, we included similar research questions that were published by different authors, which might have led to overestimation. Third, to compare effect estimates between study designs, we substituted original outcome measures, such as RR or HR instead of OR, if the number of events could not be determined from primary articles to calculate the OR, similarly to previous studies^21,44^. However, results using the RR and HR are not necessarily consistent with those using the OR, particularly when the number of events is large. Fourth, we were unable to estimate the ROR adjusted for the methodological quality of systematic reviews based on the AMSTAR 2 tool, as almost all reviews were judged to be of low quality. Fifth, in the present study, we performed a literature search using two databases recommended by AMSTAR 2: the EMBASE and MEDLINE databases, which are the most universally used in the medical field. Sixth, we were unable to adjust for several potential risk factors that may have influenced the results in each primary study, such as the sample size, details concerning the techniques used to adjust for confounding factors, presence of selection bias, degree of risk of bias, and funding sources. In particular, differences in the sample size in each primary study might have influenced the results, but we only adjusted for differences in the number of primary articles between study designs at the systematic review level. Future studies should explore those risk factors at the primary study level. In addition, meta-analyses of observational studies are likely to have dramatically increased in number over the past few years, so we must continue to update our research. Finally, because we sampled meta-analyses which included both observational studies and RCTs, it is conceivable that extreme results, either from observational studies or from RCTs, could have been excluded when the original meta-analysis was conducted, leading to spurious greater concordance between the two study designs. Without a pre-specified protocol, we cannot assess the extent of such practices.

## Conclusion

This study indicates that evidence synthesis based on observational studies has been increasing in nephrology. When we examined ROR, we found no systematic differences in effect estimates between observational studies and RCTs when meta-analyses included both study design types. These findings might encourage researchers to justify the inclusion of observational studies in meta-analyses. This approach can increase statistical power and allow stronger causal inference. However, caution is needed when interpreting the findings from both observational studies and RCTs because the interpretations based on statistical significance were shown to be less concordant than those based on ROR. Further studies are necessary to explore the causes of these contradictions.

## Supplementary Information

Supplementary Information

## Data Availability

The datasets used and/or analysed during the current study are available from the corresponding author on reasonable request.

## References

[1] Nardini C (2014). The ethics of clinical trials. Ecancermedicalscience.

[2] Black N (1996). Why we need observational studies to evaluate the effectiveness of health care. BMJ.

[3] Egger M, Schneider M, Davey SG (1998). Spurious precision? Meta-analysis of observational studies. BMJ.

[4] Barton S (2000). Which clinical studies provide the best evidence? The best RCT still trumps the best observational study. BMJ.

[5] Strippoli GF, Craig JC, Schena FP (2004). The number, quality, and coverage of randomized controlled trials in nephrology. J. Am. Soc. Nephrol..

[6] Samuels JA, Molony DA (2012). Randomized controlled trials in nephrology: State of the evidence and critiquing the evidence. Adv. Chronic Kidney Dis..

[7] Campbell MK (2000). Evidence-based medicine in nephrology: Identifying and critically appraising the literature. Nephrol. Dial. Transplant..

[8] Palmer SC, Sciancalepore M, Strippoli GF (2011). Trial quality in nephrology: How are we measuring up?. Am. J. Kidney Dis..

[9] Charytan D, Kuntz RE (2006). The exclusion of patients with chronic kidney disease from clinical trials in coronary artery disease. Kidney Int..

[10] Deo A, Schmid CH, Earley A, Lau J, Uhlig K (2011). Loss to analysis in randomized controlled trials in CKD. Am. J. Kidney Dis..

[11] Garg AX, Hackam D, Tonelli M (2008). Systematic review and meta-analysis: When one study is just not enough. Clin. J. Am. Soc. Nephrol..

[12] Norris SL (2011). Observational studies in systematic [corrected] reviews of comparative effectiveness: AHRQ and the Effective Health Care Program. J. Clin. Epidemiol..

[13] Guyatt GH (2011). GRADE guidelines: 9 rating up the quality of evidence. J. Clin. Epidemiol..

[14] Reeves BC (2013). An introduction to methodological issues when including non-randomised studies in systematic reviews on the effects of interventions. Res. Synth. Methods..

[15] Greene T (2009). Randomized and observational studies in nephrology: How strong is the evidence?. Am. J. Kidney Dis..

[16] Ray JG (2002). Evidence in upheaval: Incorporating observational data into clinical practice. Arch. Intern. Med..

[17] Klein-Geltink JE, Rochon PA, Dyer S, Laxer M, Anderson GM (2007). Readers should systematically assess methods used to identify, measure and analyze confounding in observational cohort studies. J. Clin. Epidemiol..

[18] Kuss O, Legler T, Borgermann J (2011). Treatments effects from randomized trials and propensity score analyses were similar in similar populations in an example from cardiac surgery. J. Clin. Epidemiol..

[19] Lonjon G (2014). Comparison of treatment effect estimates from prospective nonrandomized studies with propensity score analysis and randomized controlled trials of surgical procedures. Ann. Surg..

[20] Tzoulaki I, Siontis KC, Ioannidis JP (2011). Prognostic effect size of cardiovascular biomarkers in datasets from observational studies versus randomised trials: Meta-epidemiology study. BMJ.

[21] Anglemyer A, Horvath HT, Bero L (2014). Healthcare outcomes assessed with observational study designs compared with those assessed in randomized trials. Cochrane Database Syst. Rev..

[22] Sterne JA (2002). Statistical methods for assessing the in uence of study characteristics on treatment e ects in ‘meta-epidemiological’ research. Statist. Med..

[23] Liberati A (2009). The PRISMA statement for reporting systematic reviews and meta-analyses of studies that evaluate healthcare interventions: Explanation and elaboration. BMJ.

[24] Wells GA (2020). The Newcastle-Ottawa Scale (NOS) for Assessing the Quality of Nonrandomised Studies in Meta-analysis.

[25] Sterne JAC, Higgins JPT, Reeves BC. *A Cochrane Risk of Bias Assessment Tool: For Non-Randomized Studies of Interventions *(ACROBAT-NRSI), Version 1.0.0, http://www.riskofbias.info. (2014).

[26] Stroup DF (2000). Meta-analysis of observational studies in epidemiology: A proposal for reporting. Meta-analysis of observational studies in epidemiology (MOOSE) group. JAMA.

[27] Moher D, Cook DJ, Eastwood S, Olkin I, Rennie D, Stroup DF (1999). Improving the quality of reports of meta-analyses of randomised controlled trials: The QUOROM statement. Lancet.

[28] Vandenbroucke JP (2007). Strengthening the reporting of observational studies in epidemiology (STROBE): Explanation and elaboration. Epidemiology.

[29] Schulz KF, Altman DG, Moher D (2010). CONSORT 2010 statement: Updated guidelines for reporting parallel group randomised trials. BMJ.

[30] Higgins, J. P. T., Green, S. Cochrane Handbook for Systematic Reviews of Interventions Version 5.1.0 [updated March 2011]. The Cochrane Collaboration, www.cochrane-handbook.org (2011).

[31] Golder S, Loke YK, Bland M (2011). Meta-analyses of adverse effects data derived from randomised controlled trials as compared to observational studies: Methodological overview. PLoS Med..

[32] Shea BJ (2017). AMSTAR 2: A critical appraisal tool for systematic reviews that include randomised or non-randomised studies of healthcare interventions, or both. BMJ.

[33] Tajika A, Ogawa Y, Takeshima N, Hayasaka Y, Furukawa TA (2015). Replication and contradiction of highly cited research papers in psychiatry: 10-year follow-up. Br. J. Psychiatry..

[34] Sterne JA (2002). Statistical methods for assessing the influence of study characteristics on treatment effects in 'meta-epidemiological' research. Stat.Med..

[35] Deeks JJ (2003). European Carotid Surgery Trial Collaborative Group. Evaluating non-randomised intervention studies. Health Technol. Assess..

[36] Kyriacou DN, Lewis RJ (2016). Confounding by indication in clinical research. JAMA.

[37] Rothman KJ, Greenland S, Lash TL (2008). Modern Epidemiology.

[38] Patel CJ, Burford B, Ioannidis JP (2015). Assessment of vibration of effects due to model specification can demonstrate the instability of observational associations. J. Clin. Epidemiol..

[39] Ripollone JE, Huybrechts KF, Rothman KJ, Ferguson RE, Franklin JM (2018). Implications of the propensity score matching paradox in pharmacoepidemiology. Am. J. Epidemiol..

[40] Staffa SJ, Zurakowski D (2018). Five steps to successfully implement and evaluate propensity score matching in clinical research studies. Anesth. Analg..

[41] Sterne JA (2016). ROBINS-I: A tool for assessing risk of bias in non-randomised studies of interventions. BMJ.

[42] Viswanathan M, Berkman ND (2012). Development of the RTI item bank on risk of bias and precision of observational studies. J. Clin. Epidemiol..

[43] Lo CK, Mertz D, Loeb M (2014). Newcastle-Ottawa Scale: Comparing reviewers' to authors' assessments. BMC Med. Res. Methodol..

[44] Hayden JA, Cote P, Bombardier C (2006). Evaluation of the quality of prognosis studies in systematic reviews. Ann. Intern. Med..

[45] Downs SH, Black N (1998). The feasibility of creating a checklist for the assessment of the methodological quality both of randomised and non-randomised studies of health care interventions. J. Epidemiol. Commnity Health..

[46] Harris RP (2001). Current methods of the U.S. preventive services task force a review of the process. Am. J. Prev. Med..

