# Supplementary material for: Systematic differences in effect estimates between observational studies and randomized control trials in meta-analyses in nephrology
Source: Sci Rep. 2021 Mar 17;11:6088. doi: 10.1038/s41598-021-85519-5 (PMC7971062; doi:10.1038/s41598-021-85519-5)
Supplement: Supplementary file 1 — Supplementary Information [file 41598_2021_85519_MOESM1_ESM.doc]

**Systematic differences in effect estimates between observational studies and randomized control trials in meta-analyses in nephrology**

Miho Kimachi^1*^, Akira Onishi^2^, Aran Tajika^3^, Kimihiko Kimachi^1^, Toshi A Furukawa^4^

^1^ Department of Healthcare Epidemiology, School of Public Health in the Graduate School of Medicine, Kyoto University, Kyoto, Japan

^2^ Department of Rheumatology and Clinical Immunology, Kobe University Graduate School of Medicine, Hyōgo, Japan.

^3^ Department of Psychiatry, Kyoto University Hospital, Kyoto, Japan

^4^ Department of Health Promotion and Human Behavior, School of Public Health in the Graduate School of Medicine, Kyoto University, Kyoto, Japan

Japan. Health, Kyoto, Japan.

**Supplement Table 1.** Search Strategy

| **EMBASE** | |
| --- | --- |
| 1. | epidemiology/ |
| 2. | exp case control study/ |
| 3. | exp cohort analysis/ |
| 4. | (cohort adj (study or studies)).mp. |
| 5. | (Case control adj (study or studies)).mp. |
| 6. | Cohort analy$.mp. |
| 7. | exp follow up/ |
| 8. | (Follow up adj (study or studies)).mp. |
| 9. | exp observational study/ |
| 10. | observational adj (study or studies)).mp. |
| 11. | exp longitudinal study/ |
| 12. | (Longitudinal adj (study or studies)).mp. |
| 13. | exp retrospective study/ |
| 14. | (retrospective adj (study or studies)).mp. |
| 15. | exp cross-sectional study/ |
| 16. | (Cross sectional adj (study or studies)).mp. |
| 17. | or/1-16 |
| 18. | exp meta analysis/ |
| 19. | exp "Systematic Review"/ |
| 20. | (systematic$ adj2 (review$ or overview)).ti,ab. |
| 21. | (meta-analys$ or meta analys$ or metaanalys$).ti,ab. |
| 22. | (quantitativ$ adj2 (review$ or overview or synthesis$)).ti,ab. |
| 23. | (methodologic$ adj2 (review$ or overview or synthesis$)).ti,ab. |
| 24. | (integrative research review$ or research integration).ti,ab. |
| 25. | or/18-24 |
| 26. | 17 and 25 |
| 27. | exp nephrology/ |
| 28. | exp kidney disease/ |
| 29. | (Nephrolog$ or kidney$ or renal).mp. |
| 30. | or/27-29 |
| 31. | 26 and 30 |
| 32. | limit 31 to yr="2006 – 2016” |
| **MEDLINE** | |
| 1. | Epidemiologic studies/ |
| 2. | exp case control studies/ |
| 3. | exp cohort studies/ |
| 4. | (cohort adj (study or studies)).mp. |
| 5. | Case control.mp. |
| 6. | (cohort adj (study or studies)).mp. |
| 7. | Cohort analy$.mp. |
| 8. | (Follow up adj (study or studies)).mp. |
| 9. | (observational adj (study or studies)).mp. |
| 10. | Longitudinal.mp. |
| 11. | Retrospective.mp. |
| 12. | Cross sectional.mp. |
| 13. | Cross-sectional studies/ |
| 14. | or/1-13 |
| 15. | review or review,tutorial or review, academic).pt. |
| 16. | (medline or medlars or embase or pubmed or cochrane).tw,sh. |
| 17. | ((hand adj2 search$) or (manual$ adj2 search$)).tw,sh. |
| 18. | (electronic database$ or bibliographic database$ or computeried database$ or online database$).tw,sh. |
| 19. | 1or/16-18 |
| 20. | 15 and 19 |
| 21. | meta-analysis.pt. |
| 22. | meta-analysis.sh. |
| 23. | (meta-analys$ or meta analys$ or metaanalys$).tw,sh. |
| 24. | (systematic$ adj5 review$).tw,sh. |
| 25. | (systematic$ adj5 overview$).tw,sh. |
| 26. | (quantitativ$ adj5 review$).tw,sh. |
| 27. | (quantitativ$ adj5 overview$).tw,sh. |
| 28. | (quantitativ$ adj5 synthesis$).tw,sh. |
| 29. | (methodologic$ adj5 review$).tw,sh. |
| 30. | (methodologic$ adj5 overview$).tw,sh. |
| 31. | (integrative research review$ or research integration).tw. |
| 32. | or/21-31 |
| 33. | 19 or 32 |
| 34. | 14 and 33 |
| 35. | exp Nephrology/ |
| 36. | exp Kidney Diseases/ |
| 37. | (Nephrolog$ or kidney$ or renal).mp. |
| 38. | or/35-37 |
| 39. | 34 and 38 |
| 40. | limit 39 to yr="2006 - 2016" |

**Supplement Table 2-1.** Reference to studies included in or excluded from the meta-analyses to compare the effect estimates between study designs

| **References to studies included in the meta-analyses to compare the effect estimates between study designs** |
| --- |
| 1. Zhou X, *et al.* Impact of bariatric surgery on renal functions in patients with type 2 diabetes: systematic review of randomized trials and observational studies. *Surg Obes Relat Dis.* 12, 1873-1882 (2016). 2. Zhou S, Wu C, Song Q, Yang X, Wei Z. Effect of angiotensin-converting enzyme inhibitors in contrast-induced nephropathy: a meta-analysis. *Nephron.* 133, 1-14 (2016). 3. Zhao C, et al. Comparison of laparoscopic stone surgery and percutaneous nephrolithotomy in the management of large upper urinary stones: a meta-analysis. *Urolithiasis.* 44, 479-490 (2016). 4. Wu P, Wang L, Wang K. Supine versus prone position in percutaneous nephrolithotomy for kidney calculi: a meta-analysis. *Int Urol Nephrol.* 43, 67-77 (2011). 5. Wierstra BT, *et al.* The impact of "early" versus "late" initiation of renal replacement therapy in critical care patients with acute kidney injury: a systematic review and evidence synthesis. *Crit Care.* 20, 122 (2016). 6. Wang Z, *et al*. Partial nephrectomy vs. radical nephrectomy for renal tumors: a meta-analysis of renal function and cardiovascular outcomes. *Urol Oncol.* 34, 533.e11-533.e19 (2016). 7. Toth-Manikowski SM, Francis JM, Gautam A, Gordon CE. Outcomes of bisphosphonate therapy in kidney transplant recipients: a systematic review and meta-analysis. *Clin Transplant.* 30, 1090-1096 (2016). 8. Shaw C, Nitsch D, Lee J, Fogarty D, Sharpe CC. Impact of an Early Invasive Strategy versus conservative strategy for unstable angina and non-ST elevation acute coronary syndrome in patients with chronic kidney disease: a systematic review. *PLoS One.* 11, e0153478 (2016). 9. Mi Y, *et al.* Flexible ureterorenoscopy (F-URS) with holmium laser versus extracorporeal shock wave lithotripsy (ESWL) for treatment of renal stone <2 cm: a meta-analysis. *Urolithiasis.* 44, 353-365 (2016). 10. Khawaja AZ, *et al.* Systematic review of drug eluting balloon angioplasty for arteriovenous haemodialysis access stenosis. *J Vasc Access.* 17, 103-110 (2016). 11. Ipema KJ, *et al.* Nutritional status in nocturnal hemodialysis patients - a systematic review with meta-analysis. *PLoS One.* 11, e0157621 (2016). 12. Hameed AM, Pleass HC, Wong G, Hawthorne WJ. Maximizing kidneys for transplantation using machine perfusion: from the past to the future: a comprehensive systematic review and meta-analysis. *Medicine (Baltimore)*. 95, e5083 (2016). 13. Cheungpasitporn W, Rossetti S, Friend K, Erickson SB, Lieske JC. Treatment effect, adherence, and safety of high fluid intake for the prevention of incident and recurrent kidney stones: a systematic review and meta-analysis. *J Nephrol.* 29, 211-219 (2016). 14. Thongprayoon C, Cheungpasitporn W, Vijayvargiya P, Anthanont P, Erickson SB. The risk of kidney stones following bariatric surgery: a systematic review and meta-analysis. *Ren Fail.* 38, 424-430 (2016). 15. Zhang L, *et al.* Extended daily dialysis versus continuous renal replacement therapy for acute kidney injury: a meta-analysis. *Am J Kidney Dis.* 66, 322-330 (2015). 16. Yanik EL, Siddiqui K, Engels EA. Sirolimus effects on cancer incidence after kidney transplantation: a meta-analysis. *Cancer Med.* 4, 1448-1459 (2015). 17. Yang Q, Shi Y, Yang Y, Lou G, Lv F. Association between adefovir dipivoxil treatment and the risk of renal insufficiency in patients with chronic hepatitis B: a meta-analysis. *Biomed Rep.* 3, 269-275 (2015). 18. Wu Z, et al. The effect of renin-angiotensin-aldosterone system blockade medications on contrast-induced nephropathy in patients undergoing coronary angiography: a meta-analysis. *PLoS One.* 10, e0129747 (2015). 19. Wang SM, et al. Multidisciplinary care in patients with chronic kidney disease: a systematic review and meta-analysis. *Eur J Intern Med.* 26, 640-645 (2015). 20. Wang J, Gu C, Gao M, Yu W, Yu Y. Preoperative statin therapy and renal outcomes after cardiac surgery: a meta-analysis and meta-regression of 59,771 Patients. *Can J Cardiol.* 31, 1051-1060 (2015). 21. Velazquez N, *et al.* Medical expulsive therapy for pediatric urolithiasis: systematic review and meta-analysis. *J Pediatr Urol.* 11, 321-327 (2015). 22. Tian ML, Hu Y, Yuan J, Zha Y. Efficacy and safety of perioperative sodium bicarbonate therapy for cardiac surgery-associated acute kidney injury: a meta-analysis. *J Cardiovasc Pharmacol.* 65, 130-136 (2015). 23. Thongprayoon C, *et al.* Acute kidney injury after transcatheter aortic valve replacement: a systematic review and meta-analysis. *Am J Nephrol.* 41, 372-382 (2015). 24. Thamcharoen N, Thongprayoon C, Edmonds PJ, Cheungpasitporn W. Periprocedural nebivolol for the prevention of contrast-induced acute kidney injury: a systematic review and meta-analysis. *N Am J Med Sci.* 7, 446-451 (2015). 25. Sarathy H, *et al.* The effects of short-term vitamin D supplementation on glucose metabolism in dialysis patients: a systematic review and meta-analysis. *Int Urol Nephrol*. 47, 537-549 (2015). 26. Liu LL, *et al*. Tonsillectomy for IgA nephropathy: a meta-analysis. *Am J Kidney Dis.* 80-87 (2015). 27. Hanrahan T, Whitehouse T, Lipman J, Roberts JA. Vancomycin-associated nephrotoxicity: a meta-analysis of administration by continuous versus intermittent infusion. *Int J Antimicrob Agents.* 46, 249-253 (2015). 28. Fu N, *et al.* A meta-analysis of stent placement vs. angioplasty for dialysis vascular access stenosis. *Semin Dial.* 28, 311-317 (2015). 29. Cooper J, Power AH, DeRose G, Forbes TL, Dubois L. Similar failure and patency rates when comparing one- and two-stage basilic vein transposition. *J Vasc Surg.*61, 809-816 (2015). 30. Cheungpasitporn W, et al. Preoperative renin-angiotensin system inhibitors use linked to reduced acute kidney injury: a systematic review and meta-analysis. *Nephrol Dial Transplant.* 30, 978-988 (2015). 31. Bolignano D, D'Arrigo G, Pisano A, Coppolino G. Pentoxifylline for anemia in chronic kidney disease: a systematic review and meta-analysis. *PLoS One.* 10, e0134104 (2015). 32. Bashar K, *et al*. One-stage vs. two-stage brachio-basilic arteriovenous fistula for dialysis access: a systematic review and a meta-analysis. *PLoS One.* 10, e0120154 (2015). 33. Zhang XL, *et al.* Statin use and risk of kidney cancer: a meta-analysis of observational studies and randomized trials. *Br J Clin Pharmacol.* 77, 458-465 (2014). 34. Pan SY, *et al.* Effect of preoperative statin therapy on postoperative acute kidney injury in patients undergoing major surgery: systemic review and meta-analysis. *Nephrology (Carlton).* 19, 750-763 (2014). 35. Liu Y, Davari-Farid S, Arora P, Porhomayon J, Nader ND. Early versus late initiation of renal replacement therapy in critically ill patients with acute kidney injury after cardiac surgery: a systematic review and meta-analysis. *J Cardiothorac Vasc Anesth.* 28, 557-563 (2014). 36. Katsanos K, *et al.* Systematic review and meta-analysis of thermal ablation versus surgical nephrectomy for small renal tumours. *Cardiovasc Intervent Radiol.* 37, 427-437 (2014). 37. Alberts VP, Idu MM, Legemate DA, Laguna Pes MP, Minnee RC. Ureterovesical anastomotic techniques for kidney transplantation: a systematic review and meta-analysis. *Transpl Int*. 27, 593-605 (2014). 38. Wang ZJ, *et al*. Drug-eluting stents versus bare-metal stents in patients with decreased GFR: a meta-analysis. *Am J Kidney Dis.* 62, 711-721 (2013). 39. Schneider AG, *et al.* Choice of renal replacement therapy modality and dialysis dependence after acute kidney injury: a systematic review and meta-analysis. *Intensive Care Med.* 39, 987-997 (2013). 40. Jin HM, Guo LL, Zhan XL, Pan Y. Effect of prolonged weekly hemodialysis on survival of maintenance hemodialysis patients: a meta-analysis of studies. *Nephron Clin Pract.* 123, 220-228 (2013). 41. Hagen SM, Lafranca JA, Steyerberg EW, Ĳzermans JN, Dor FJ. Laparoscopic versus open peritoneal dialysis catheter insertion: a meta-analysis. *PLoS One.*8, e56351 (2013). 42. Fan X, *et al*. Comparison of transperitoneal and retroperitoneal laparoscopic nephrectomy for renal cell carcinoma: a systematic review and meta-analysis. *BJU Int*. 111, 611-621(2013). 43. Deo SV, *et al.* Myocardial revascularisation in renal dysfunction: a systematic review and meta-analysis. *Heart Lung Circ.* 22, 827-835 (2013). 44. Davis MI, *et al*. Effectiveness of renal denervation therapy for resistant hypertension: a systematic review and meta-analysis. *J Am Coll Cardiol.* 62, 231-241 (2013). 45. Chionh CY, Soni SS, Finkelstein FO, Ronco C, Curz DN. Use of peritoneal dialysis in AKI: a systematic review. *Clin J Am Soc Nephrol.* 8, 1649-1660 (2013). 46. Almeida CC, *et al.* Safety of immunosuppressive drugs used as maintenance therapy in kidney transplantation: a systematic review and meta-analysis. *Pharmaceuticals (Basel).* 6, 1170-1194 (2013). 47. Bathini V, McGregor T, McAlister VC, Luke PPW, Sener A. Renal perfusion pump vs cold storage for donation after cardiac death kidneys: a systematic review. *J Urol.* 189, 2214-2220 (2013). 48. Wang X, Yuan WJ. Timing of initiation of renal replacement therapy in acute kidney injury: a systematic review and meta-analysis. *Ren Fail.* 34, 396-402 (2012). 49. Slagt IKB, Klop KWJ, Ijzermans JNM, Terkivatan T. Intravesical versus extravesical ureteroneocystostomy in kidney transplantation: a systematic review and meta-analysis. *Transplantation.* 94, 1179-1184 (2012). 50. Pan Y, Xu XD, Guo LL, Cai LL, Jin HM. Association of early versus late initiation of dialysis with mortality: systematic review and meta-analysis. *Nephron Clin Pract.* 120, c121-131 (2012). 51. Karvellas CJ, *et al.* A comparison of early versus late initiation of renal replacement therapy in critically ill patients with acute kidney injury: a systematic review and meta-analysis. *Crit Care*. 15, R72 (2011). 52. Liu L, Zheng S, Xu Y, Wei Q. Systematic review and meta­analysis of percutaneous nephrolithotomy for patients in the supine versus prone position. *J Endourol.* 24, 1941-1946 (2010). 53. Asrani SK, *et al.* Use of sirolimus in liver transplant recipients with renal insufficiency: a systematic review and meta-analysis. *Hepatology.* 52, 1360-1370 (2010). 54. Nigwekar SU, Kandula P, Hix JK, Thakar CV. Off-pump coronary artery bypass surgery and acute kidney injury: a meta-analysis of randomized and observational studies. *Am J Kidney Dis.* 54, 413-423 (2009). 55. Murad MH, *et al.* Autogenous versus prosthetic vascular access for hemodialysis: a systematic review and meta-analysis. *J Vasc Surg.* 48, 34S-47S (2008). 56. Thomas G, *et al.* Insulin therapy and acute kidney injury in critically ill patients a systematic review. *Nephrol Dial Transplant.* 22, 2849-2855 (2007). |
| **References to studies excluded from the meta-analyses to compare the effect estimates between study designs** |
| 1. Xia X, *et al.* Serum uric acid and mortality in chronic kidney disease: A systematic review and meta-analysis. *Metabolism.* 65, 1326-1341 (2016). 2. Wang H, Man L, Li G, Huang G, Liu N. Association between serum vitamin D levels and the risk of kidney stone: evidence from a meta-analysis. *Nutr J.* 15, 32 (2016). 3. Tullavardhana T, Akranurakkul P, Ungkitphaiboon W, Songtish D. Surgical versus percutaneous techniques for peritoneal dialysis catheter placement: a meta-analysis of the outcomes. *Ann Med Surg (Lond).* 10, 11-28 (2016). 4. Terpstra ML, Singh R, Geerlings SE, Bemelman FJ. Measurement of the intestinal permeability in chronic kidney disease. *World J Nephrol.* 5, 378-388 (2016). 5. Ramesh S, *et al.* Hormone therapy and clinical and surrogate cardiovascular endopoints in women with chronic kidney disease: a systematic review and meta-analysis. *Menopause.* 23, 1028-1037 (2016). 6. Pierorazio PM, *et al.* Management of Renal Masses and Localized Renal Cancer: Systematic Review and Meta-Analysis. *J Urol.* 196, 989-999 (2016). 7. Devoe DJ, *et al.* Patient education and peritoneal dialysis modality selection: a systematic review and meta-analysis. *Am J Kidney Dis*. 68, 422-433 (2016). 8. Chan HL, Shaikh J, Gupta S, Hamed K. Renal function in nucleos(t)ide analog-treated patients with chronic hepatitis B: a systematic literature review and network meta-analysis. *Adv Ther.* 33, 862-875 (2016). 9. Chen Y, Shao Y, Xu J. The survival and complication rates of laparoscopic versus open catheter placement in peritoneal dialysis patients: a meta-analysis. *Surg Laparosc Endosc Percutan Tech.* 25, 440-443 (2015). 10. Yang B, *et al.* Non-pharmacological interventions for improving sleep quality in patients on dialysis: systematic review and meta-analysis. *Sleep Med Rev.* 23, 68-82 (2015). 11. Sanguankeo A, Upala S, Cheungpasitporn W, Ungprasert P, Knight EL. Effects of statins on renal outcome in chronic kidney disease patients: a systematic review and meta-analysis. *PLoS One.* 10, e0132970 (2015). 12. Salerno F, Navickis RJ, Wilkes MM. Albumin treatment regimen for type 1 hepatorenal syndrome: a dose-response meta-analysis. *BMC Gastroenterol.* 15, 167 (2015). 13. Masson P, *et al.* Chronic kidney disease and the risk of stroke: a systematic review and meta-analysis. *Nephrol Dial Transplant.* 30, 1162-1169 (2015). 14. Derakhshanian H, Shab-Bidar S, Speakman JR, Nadimi H, Djafarian K. Vitamin D and diabetic nephropathy: a systematic review and meta-analysis. *Nutrition.* 31, 1189-1194 (2015). 15. Chokhandre MK, Mahmoud MI, Hakami T, Jafer M, Inamdar AS. Vitamin D & its analogues in type 2 diabetic nephropathy: a systematic review. *J Diabetes Metab Disord.* 14, 58 (2015). 16. Chen J, Zhou QY, Wang JD. Comparison between subtotal parathyroidectomy and total parathyroidectomy with autotransplantation for secondary hyperparathyroidism in patients with chronic renal failure: a meta-analysis. *Horm Metab Res.* 47, 643-651 (2015). 17. Chao-yang C, Xuai-yan S, Ying Z, Xia Z, Yi-min C. Efficacy and safety of tacrolimus for treating idiopathic nephrotic syndrome: a meta-analysis. *Chinese Journal of New Drugs.* 24, 64-73 (2015). 18. Asghar W, Jamali F. The effect of COX-2-selective meloxicam on the myocardial, vascular and renal risks: a systematic review. *Inflammopharmacol.* 23, 1-16 (2015). 19. Stanifer JW, et al. The epidemiology of chronic kidney disease in sub-Saharan Africa: a systematic review and meta-analysis. *The Lancet Global Health.* 2, e174-81 (2014). 20. Xing S, Yang J, Zhang X, Zhou P. Comparative efficacy and safety of mizoribine with mycophenolate mofetil for Asian renal transplantation--a meta-analysis. *Clin Biochem.* 47, 663-669 (2014). 21. Wong B, *et al.* Buttonhole versus rope-ladder cannulation of arteriovenous fistulas for hemodialysis: a systematic review. *Am J Kidney Dis.* 64, 918-936 (2014). 22. Muir CA, *et al*. Buttonhole cannulation and clinical outcomes in a home hemodialysis cohort and systematic review. *Clin J Am Soc Nephrol.* 9, 110-119 (2014). 23. Liu N, Wazir R, Wang J, Wang KJ. Maximizing the donor pool: left versus right laparoscopic live donor nephrectomy--systematic review and meta-analysis. *Int Urol Nephrol.*46, 1511-1519 (2014). 24. Kwok CS, *et al.* Renal denervation and blood pressure reduction in resistant hypertension: a systematic review and meta-analysis. *Open Heart.* 1, e000092 (2014). 25. Hill CJ, *et al.* Glycated hemoglobin and risk of death in diabetic patients treated with hemodialysis: a meta-analysis. *Am J Kidney Dis.* 63, 84-94 (2014). 26. Fleeman N, *et al.* Allopurinol for the treatment of chronic kidney disease: a systematic review. *Health Technol Assess.* 18, 1-77, v-vi (2014). 27. Zimmerman DL, *et al.* Dialysate calcium concentration and mineral metabolism in long and long-frequent hemodialysis: a systematic review and meta-analysis for a Canadian Society of Nephrology clinical practice guideline. *Am J Kidney Dis.* 62, 97-111 (2013). 28. Moos SI, van Vemde DN, Stoker J, Bipat S. Contrast induced nephropathy in patients undergoing intravenous (IV) contrast enhanced computed tomography (CECT) and the relationship with risk factors: a meta-analysis. *Eur J Radiol.* 82, e387-399 (2013). 29. Ho ET, Wong G, Craig JC, Chapman JR. Once-daily extended-release versus twice-daily standard-release tacrolimus in kidney transplant recipients: a systematic review. *Transplantation.* 95, 1120-1128 (2013). 30. Hiremath SB, Srinivas LD. Survival benefits of terlipressin and non-responder state in hepatorenal syndrome: a meta-analysis. *Indian J Pharmacol.* 45, 54-60 (2013). 31. Garg N, *et al*. Cardiac resynchronization therapy in CKD: a systematic review. *Clin J Am Soc Nephrol.* 8, 1293-1303 (2013). 32. Xie H, Zhang W, Cheng J, He Q. Laparoscopic versus open catheter placement in peritoneal dialysis patients: a systematic review and meta-analysis. *BMC Nephrol.* 13, 69 (2012). 33. Wyld M, Morton RL, Hayen A, Howard K, Webster AC. A systematic review and meta-analysis of utility-based quality of life in chronic kidney disease treatments. *PLoS Med.* 9, e1001307 (2012). 34. Susantitaphong P, Koulouridis I, Balk EM, Madias NE, Jaber BL. Effect of frequent or extended hemodialysis on cardiovascular parameters: a meta-analysis. *Am J Kidney Dis.* 59, 689-699 (2012). 35. Susantitaphong P, *et al.* GFR at initiation of dialysis and mortality in CKD: a meta-analysis. *Am J Kidney Dis.* 59, 829-840 (2012). 36. Rodriguez-Peralvarez M,*et al.* Tacrolimus trough levels, rejection and renal impairment in liver transplantation: a systematic review and meta-analysis. *Am J Transplant.* 12, 2797-2814 (2012). 37. Feng B, *et al.* Effect of interferon-alpha-based antiviral therapy on hepatitis C virus-associated glomerulonephritis: a meta-analysis. *Nephrol Dial Transplant.* 27, 640-646 (2012). 38. Fabrizi F, Dixit V, Martin P, Messa P. Erythropoietin use and immunogenicity of hepatitis B virus vaccine in chronic kidney disease patients: a meta-analysis. *Kidney Blood Press Res.* 35, 504-510 (2012). 39. Corapi KM, Chen JL, Balk EM, Gordon CE. Bleeding complications of native kidney biopsy: a systematic review and meta-analysis. *Am J Kidney Dis.* 60, 62-73 (2012). 40. Zhang T, Shen LH, Hu LH, He B. Statins for the prevention of contrast-induced nephropathy: a systematic review and meta-analysis. *Am J Nephrol.* 33, 344-351 (2011). 41. Mafham M, Emberson J, Landray MJ, Wen CP, Baigent C. Estimated glomerular filtration rate and the risk of major vascular events and all-cause mortality: a meta-analysis. *PLoS One.* 6, e25920 (2011). 42. Kandula P, *et al.* Vitamin D supplementation in chronic kidney disease: a systematic review and meta-analysis of observational studies and randomized controlled trials. *Clin J Am Soc Nephrol.* 6, 50-62 (2011). 43. Currie AC, Knight SR, Morris PJ. Tuberculosis in renal transplant recipients: the evidence for prophylaxis. *Transplantation.* 90, 695-704 (2010). 44. Afshinnia F, Wilt TJ, Duval S, Esmaeili A, Ibrahim HN. Weight loss and proteinuria: systematic review of clinical trials and comparative cohorts. *Nephrol Dial Transplant.* 25, 1173-1183 (2010). 45. Yarlagadda SG, Coca SG, Formica RN Jr, Poggio ED, Parikh CR. Association between delayed graft function and allograft and patient survival: a systematic review and meta-analysis. *Nephrol Dial Transplant.* 24, 1039-1047 (2009). 46. Navaneethan SD, *et al.* Weight loss interventions in chronic kidney disease: a systematic review and meta-analysis. *Clin J Am Soc Nephrol.* 4, 1565-1574 (2009). 47. Heinz J, Kropf S, Luley C, Dierkes J. Homocysteine as a risk factor for cardiovascular disease in patients treated by dialysis: a meta-analysis. *Am J Kidney Dis.* 54, 478-489 (2009). 48. Bonafont X, *et al.* A meta-analysis of the relative doses of erythropoiesis-stimulating agents in patients undergoing dialysis. *NDT Plus.* 2, 347-353 (2009). 49. Seabra VF, *et al.* Timing of renal replacement therapy initiation in acute renal failure: a meta-analysis. *Am J Kidney Dis.* 52, 272-284 (2008). 50. Pannu N, *et al.* Renal replacement therapy in patients with acute renal failure: a systematic review. *JAMA*. 299, 793-805 (2008). 51. Gordon CE, *et al.* Interferon treatment in hemodialysis patients with chronic hepatitis C virus infection: a systematic review of the literature and meta-analysis of treatment efficacy and harms. *Am J Kidney Dis.* 51, 263-277 (2008). 52. Damman K, *et al*. Worsening renal function and prognosis in heart failure: systematic review and meta-analysis. *J Card Fail.* 13, 599-608 (2007). 53. Coca SG, Peixoto AJ, Garg AX, Krumholz HM, Parikh CR. The prognostic importance of a small acute decrement in kidney function in hospitalized patients: a systematic review and meta-analysis. *Am J Kidney Dis*. 50, 712-720 (2007). 54. Tonelli M, *et al*. Chronic kidney disease and mortality risk: a systematic review. *J Am Soc Nephrol.* 17, 2034-2047 (2006). 55. Smith GL, *et al.* Renal impairment and outcomes in heart failure: systematic review and meta-analysis. *J Am Coll Cardiol.* 47, 1987-1996 (2006). 56. Mulay AV, Cockfield S, Stryker R, Fergusson D, Knoll GA. Conversion from calcineurin inhibitors to sirolimus for chronic renal allograft dysfunction: a systematic review of the evidence. *Transplantation.* 82, 1153-1162 (2006). 57. Moore RA, Derry S. Systematic review and meta-analysis of randomised trials and cohort studies of mycophenolate mofetil in lupus nephritis. *Arthritis Res Ther.* 8, R182 (2006). 58. Lim W, Dentali F, Eikelboom JW, Crowther MA. Meta-analysis: low-molecular-weight heparin and bleeding in patients with severe renal insufficienecy. *Ann Intern Med*. 144, 673-684 (2006). |

**Supplement Table 2-2.** Characteristics of excluded studies

| **Study** | **Reason for exclusion** |
| --- | --- |
| Xia 2016 | not interventional studies |
| Wang 2016 | not interventional studies |
| Tullavardhana 2016 | not included randomized control trials in meta-analyses |
| Terpstra 2016 | not interventional studies |
| Ramesh 2016 | not specific research questions |
| Pierorazio 2016 | not specific research questions |
| Devoe 2016 | included before-after studies in meta-analyses |
| Chan 2016 | network meta-analyses |
| Chen 2015 | not specific research questions |
| Yang 2015 | not included observational studies in meta-analyses |
| Sanguankeo 2015 | not included observational studies in meta-analyses |
| Salerno 2015 | multiple comparisons |
| Masson 2015 | not interventional studies |
| Derakhshanian 2015 | not specific research questions |
| Chokhandre 2015 | included before-after studies in meta-analyses |
| Chen 2015 | not included randomized control trials in meta-analyses |
| Chao-yang 2015 | included case series studies in meta-analyses |
| Asghar 2015 | multiple comparisons |
| Stanifer 2014 | not interventional studies |
| Xing 2014 | unclear data of primary studies |
| Wong 2014 | included before-after studies in meta-analyses |
| Muir 2014 | included before-after studies in meta-analyses |
| Liu 2014 | not included randomized control trials in meta-analyses |
| Kwok 2014 | not included observational studies in meta-analyses |
| Hill 2014 | not interventional studies |
| Fleeman 2014 | not included observational studies in meta-analyses |
| Zimmerman 2013 | not specific research questions |
| Moos 2013 | not specific research questions |
| Ho 2013 | not specific research questions |
| Hiremath 2013 | inappropriate effect indicators |
| Garg 2013 | not specific research questions |
| Xie 2012 | not specific research questions |
| Wyld 2012 | not specific research questions |
| Susantitaphong 2012 | included single arm studies in meta-analyses |
| Susantitaphong 2012 (2) | not interventional studies |
| Rodriguez-Peralvarez 2012 | not included observational studies in meta-analyses |
| Feng 2012 | not included randomized control trials in meta-analyses |
| Fabrizi 2012 | unclear data of primary studies |
| Corapi 2012 | inappropriate effect indicators |
| Zhang 2011 | not included observational studies in meta-analyses |
| Mafham 2011 | not interventional studies |
| Kandula 2011 | included before-after studies in meta-analyses |
| Currie 2010 | not included observational studies in meta-analyses |
| Afshinnia 2010 | included single arm studies in meta-analyses |
| Yarlagadda 2009 | not interventional studies |
| Navaneethan 2009 | included before-after studies in meta-analyses |
| Heinz 2009 | not specific research questions |
| Bonafont 2009 | inappropriate effect indicators |
| Seabra 2008 | included single arm studies in meta-analyses |
| Pannu 2008 | not included observational studies in meta-analyses |
| Gordon 2008 | inappropriate effect indicators |
| Damman 2007 | not interventional studies |
| Coca 2007 | not interventional studies |
| Tonelli 2006 | not interventional studies |
| Smith 2006 | not interventional studies |
| Mulay 2006 | included single arm studies in meta-analyses |
| Moore 2006 | inappropriate effect indicators |
| Lim 2006 | inappropriate effect indicators |

**Supplement Table 3.** Trends in the number of published meta-analyses of observational studies over the past decades by country

|  | 2006 | 2007 | 2008 | 2009 | 2010 | 2011 | 2012 | 2013 | 2014 | 2015 | 2016 | Total |
| --- | --- | --- | --- | --- | --- | --- | --- | --- | --- | --- | --- | --- |
| China | 0 | 0 | 1 | 1 | 2 | 9 | 15 | 35 | 35 | 63 | 51 | 212 |
| Other Asian countries | 0 | 0 | 0 | 0 | 0 | 1 | 1 | 1 | 2 | 3 | 3 | 11 |
| USA | 3 | 4 | 5 | 6 | 8 | 3 | 8 | 6 | 9 | 12 | 22 | 86 |
| Canada | 4 | 2 | 2 | 1 | 2 | 2 | 1 | 7 | 5 | 4 | 2 | 32 |
| UK | 1 | 2 | 1 | 3 | 2 | 1 | 2 | 1 | 7 | 4 | 6 | 30 |
| Italy | 0 | 0 | 1 | 0 | 0 | 1 | 1 | 1 | 5 | 7 | 3 | 19 |
| Netherland | 0 | 0 | 0 | 0 | 0 | 0 | 2 | 4 | 1 | 0 | 2 | 9 |
| Other European countries | 0 | 1 | 0 | 4 | 1 | 3 | 4 | 1 | 4 | 4 | 7 | 29 |
| Australia | 0 | 1 | 1 | 2 | 0 | 2 | 2 | 3 | 2 | 2 | 4 | 19 |
| NZ | 0 | 0 | 0 | 0 | 0 | 1 | 0 | 1 | 0 | 1 | 0 | 3 |
| Brazil | 0 | 0 | 1 | 0 | 0 | 0 | 1 | 2 | 2 | 3 | 0 | 9 |
| Other Latin America | 1 | 0 | 0 | 0 | 0 | 0 | 0 | 0 | 1 | 1 | 0 | 3 |
| Middle East/Africa | 0 | 0 | 0 | 0 | 0 | 1 | 0 | 0 | 2 | 2 | 1 | 6 |
| Southeast Asia | 0 | 0 | 0 | 2 | 0 | 0 | 0 | 3 | 0 | 0 | 4 | 9 |
|  | 9 | 10 | 12 | 19 | 15 | 24 | 37 | 65 | 75 | 106 | 105 | 477 |

USA, United States of America; UK, United Kingdom; NZ, New Zealand

**Supplement Table 4.** Rating of critical domains of the assessment of multiple systematic reviews (AMSTAR) tool

|  | total | No discrepancy (significant in the same direction) (n=14) | No discrepancy (both non-significant) (n=15) | Discrepancy  (one significant and the other non-significant) (n=27) |
| --- | --- | --- | --- | --- |
| Assessment of AMSTAR appraisal tool |  |  |  |  |
| Protocol registered before commencement of the review (item 2) |  |  |  |  |
| Yes | 1 (1.8) | 0 (0) | 1 (6.7) | 0 (0) |
| Partial Yes | 3 (5.4) | 2 (14.3) | 1 (6.7) | 0 (0) |
| No | 52 (92.9) | 12 (85.7) | 13 (86.7) | 27 (100) |
| Adequacy of the literature search (item 4) |  |  |  |  |
| Yes | 0 (0) | 0 (0) | 0 (0) | 0 (0) |
| Partial Yes | 31 (55.4) | 7 (50.0) | 11 (73.3) | 13 (48.2) |
| No | 25 (44.6) | 7 (50.0) | 4 (26.7) | 14 (51.9) |
| Justification for excluding individual studies (item 7) |  |  |  |  |
| Yes | 3 (5.4) | 1 (7.1) | 1 (6.7) | 1 (3.7) |
| Partial Yes | 0 (0) | 0 (0) | 0 (0) | 0 (0) |
| No | 53 (94.6) | 13 (92.9) | 14 (93.3) | 26 (96.3) |
| Risk of bias from individual studies being included in the review (item 9) |  |  |  |  |
| *RCT* |  |  |  |  |
| Yes | 17 (30.4) | 6 (42.9) | 3 (20.0) | 8 (29.6) |
| Partial Yes | 8 (14.3) | 2 (14.3) | 1 (6.7) | 5 (18.5) |
| No | 31 (55.4) | 6 (42.9) | 11 (73.3) | 14 (51.9) |
| *NRSI* |  |  |  |  |
| Yes | 2 (3.6) | 0 (0) | 0 (0) | 2 (7.4) |
| Partial Yes | 33 (58.9) | 9 (64.3) | 9 (60.0) | 15 (55.6) |
| No | 21 (37.5) | 5 (35.7) | 6 (40.0) | 10 (37.0) |
| Appropriateness of meta-analytical methods (item 11) |  |  |  |  |
| *RCTs -yes* | 34 (60.7) | 9 (64.3) | 8 (53.3) | 17 (63.0) |
| *NRSI -yes* | 6 (10.7) | 0 (0) | 3 (20.0) | 3 (11.1) |
| Consideration of risk of bias when interpreting the results of the review (item 13) -yes | 5 (8.9) | 1 (7.1) | 0 (0) | 4 (14.8) |
| Assessment of presence and likely impact of publication bias (item 15) -yes | 33 (58.9) | 10 (71.4) | 9 (60.0) | 14 (51.9) |
| Rating overall confidence in the results of the review |  |  |  |  |
| High | 0 (0) | 0 (0) | 0 (0) | 0 (0) |
| Moderate | 0 (0) | 0 (0) | 0 (0) | 0 (0) |
| Low | 3 (5.4) | 0 (0) | 0 (0) | 3 (11.1) |
| Critically low | 53 (94.6) | 100 (100) | 100 (100) | 24 (88.9) |
